# Psychosis, Telehealth, and COVID-19: Successes and Lessons Learned From the First Wave of the Pandemic

**DOI:** 10.1017/dmp.2021.42

**Published:** 2021-02-16

**Authors:** Serena Chaudhry, Ashley Weiss, Grinasha Dillon, Ariana O’Shea, Tonya Cross Hansel

**Affiliations:** 1 Early Psychosis Intervention Clinic, New Orleans (EPIC-NOLA), LA, USA; 2 Department of Psychiatry and Behavioral Services, Tulane University School of Medicine, New Orleans, LA, USA; 3 The School of Social Work, Tulane University, New Orleans, LA, USA

**Keywords:** community mental health, mental disorders, pandemic

## Abstract

**Objective:**

This brief report analyzes a first-episode psychosis (FEP) clinic’s shift from in-person treatment to the provision of services through telemental health during the 2019 coronavirus disease (COVID-19) pandemic. The feasibility of using this technology was examined by assessing client engagement.

**Methods:**

The authors created and implemented procedures for the clinic’s transition to telemental health. Once clients’ consents were obtained, the Health Insurance Portability and Accountability Act (HIPAA) compliant platform was used to continue service provision.

**Results:**

Client engagement during this period improved compared to that of the same quarter in the previous year. Telemental health was also practical for providing groups and other supportive services to meet clients’ needs.

**Conclusion:**

Telemental health is an effective approach to providing care at an FEP clinic during a pandemic. Successes and lessons learned from the first wave of the pandemic can be used to prevent an uptick in symptoms and sustain engagement for this vulnerable population during the anticipated second wave.

## Introduction

The 2019 coronavirus disease (COVID-19) pandemic continues to stress health care systems globally, with 6.9 million cases worldwide and over 1 million in the United States.^1^ Louisiana has been especially impacted by this pandemic, having one of the highest number of deaths per capita in the United States: 30.2 deaths per 100 000.^2^ Concurrently, Louisiana has one of the highest rates of mental illness in the United States.^2^ Because people with chronic mental illness seem to be more susceptible to pandemic-related stress,^3^ local mental health care providers are preparing for a sustained spike in the needs of those with mental illness. Drastic measures have been implemented throughout the country to reduce coronavirus transmission, and traditional routes for health care services have been fractured. Systems have been forced to adapt in order to meet the needs of their patients and for continuity of care. Disruption in care, specifically for people living with severe mental illness, is concerning due to its effects on symptom exacerbation and relapse.^4^

At the Early Psychosis Intervention Clinic in New Orleans (EPIC-NOLA), Louisiana, a multidisciplinary team provides comprehensive care for adolescents and young adults who first experience psychosis. EPIC’s population is unique because the city of New Orleans is more vulnerable to community transmission of COVID-19 as a result of multiple factors, including a reliance on tourism, cultural norms such as festivals and street parades, and socioeconomic challenges such as poverty.

Disengagement from treatment is very concerning for young people experiencing the early stages of psychosis, and data demonstrate that the dropout rate can reach 80% during the first year of care.^5^ Poor engagement, such as missed clinic appointments and early dropouts, impedes the detection of early symptoms of psychosis and may lead to hospitalizations.^6,7^ A 2018 scoping review found videoconferencing to be a feasible treatment solution for individuals with schizophrenia-spectrum disorders.^8^ Recently, telemental health has gained attention for improving engagement in coordinated specialty care (CSC) clinics like EPIC-NOLA.

Massive expansion and implementation of telemental health services for EPIC-NOLA were needed to reproduce the multiple services offered in the clinic’s office setting. This brief report presents the clinic’s process, ideas about expanding online services for patients, and lessons learned from the pandemic’s first wave.

## Methods

On March 13, 2020, clinic staff met to discuss transitioning to virtual care in the context of the COVID-19 pandemic. Draft procedures pertaining to a full clinic shutdown included dissemination of consent for telemental health services; instructions for downloading videoconference software; identification of patients without access to a smartphone, tablet, or computer with audio and visual capabilities, as well as a data plan or Internet connection; implementation of a daily 8:30 AM meeting (“huddle”) to review acute needs/issues (increase from 3 days/week huddle); and enactment of virtual treatment team meetings. On March 16, 2020, the clinic transitioned its psychotherapy and medication management services to telemental health, as well as its clinical meetings (treatment team meetings, coordination of care meetings, and huddle). All existing patients received text messages detailing the transition along with an electronic version of the telemental health consent form. Clinicians facilitated the downloading of the videoconferencing application, explained the process of sending a link for patients to join clinical sessions, and discussed the need for private space, as defined by the patient, during clinical visits. Providers used Tulane University School of Medicine Department of Psychiatry’s Health Insurance Portability and Accountability Act (HIPAA)-compliant version of Zoom to connect with patients.

## Results

EPIC-NOLA saw 137 patients with a total of 545 patient encounters between March 16 and May 15, 2020, New Orleans COVID-19 shelter-at-home period, compared to107 patients with 533 patient encounters during that same period in 2019. A chi-square test was conducted to assess engagement for 2020 compared to 2019. In Table 1, the cross tabulation demonstrates that the chi-square was significant, χ^2^ = 4.07, *P* = 0.049 (McNemar’s test χ^2^ = 102.7, *P* < 0.001). Results suggest that an increased proportion of visits was kept from 2019 (67%) to 2020 (72%). During the COVID-19 shelter-at-home period, the no-show rate was 28%, whereas the same period of 2019 had a no-show rate of 32%. Of the 545 patient encounters for the shelter-at-home period in 2020, 411 were for individual psychotherapy and 134 were for medication management (Table 2).

**Table 1.** Chi-square on engagement (kept visit vs no-show)

	Engagement		
	Kept Visit	No-Show	Totals
**2019**	533 (67%)	257 (32%)	790
**2020**	545 (72%)	210 (28%)	755
**Totals**	1078	467	1545

**Table 2.** Overall clinical engagement with EPIC-NOLA (pre-COVID-19 vs COVID-19)

Variable	Pre-COVID-19 March 16 to May 15, 2019 (%)	COVID-19 March 16 to May 15, 2020 (%)
Total patients seen Show-rates	107	137
Total appointments	67.47	72.18
Physician (MD) show-rate	64.73	58.51
Therapist show-rate	68.67	83.84
Hospitalization	12.00	18.00
Race		
African American (83)	59.66	59.63
Caucasian (41)	29.64	33.76
Hispanic (10)	7.32	5.50
Vietnamese (2)	0.37	0.55
American Indian (1)	3.00	0.55
Gender		
Female	29.99	29.99
Male	70.07	70.07
Engagement		
Client’s engaged > 2 years	45.40	62.20
Client’s engaged < 2 years	54.60	37.80

Patient engagement varied based on how long patients have been active with the clinic; patients within the first year of treatment had the highest number of encounters between March 16 and May 15, 2020. The hospitalization rate for this period was 13%; 18 individuals were hospitalized once, and 5 individuals had multiple hospitalizations. EPIC-NOLA’s hospitalization rate pre-COVID-19 was 11%. A Z-score relative proportion test was conducted to assess hospitalizations for 2020 compared to those of 2019. Results demonstrate marginal significance, *Z* = −1.57, *P* = 0.058, and suggest a potential trend increase in hospitalizations from 11% to 13% in 2019 and 2020, respectively.

## Discussion

Patient engagement at EPIC-NOLA is the primary measure of the clinic’s successful transition to telemental health. The 5% increase in show rate for the period 2019 to 2020 is largely due to the elimination of transportation as a barrier to clinical care. Poverty and limited public transportation are pervasive problems across Louisiana and have proven to negatively impact patient care at the clinic. Engagement of patients who were at the clinic beyond the traditional 2-year mark is also notable, suggesting that telemental health may be a low barrier clinical intervention for expanding and sustaining continuity of care. EPIC-NOLA has operated as a continuity clinic since its inception, mainly due to the inadequate number of psychiatrists and mental health clinics in New Orleans. Clinicians capitalized on the use of telemental health to learn more about patients, meet family members, get to know patients’ physical environments, and observe patients’ moods and affect in their home environments.

During the second month of quarantine, the clinic transitioned group therapy, including moms’ group, a meditation series, and wellness coaching to telemental health. The moms’ group, which had been meeting for approximately 6 months before the pandemic, connects and supports the caregivers of young people experiencing their first episode of psychosis. More than 20 different moms have attended the group since its beginning. The pre-pandemic attendance average of 2 to 6 moms remained consistent on telehealth.

A 6-week, in-person meditation group was implemented at the clinic prior to COVID-19. The clinic restarted the group during the pandemic to augment patients’ skills for managing stress and anxiety resulting from patients’ vulnerability to pandemic-related stress. Group attendance for meditation was small prior to the pandemic and remained small, ranging from 1 to 2 people weekly.

Finally, clinic staff were contemplating a wellness program prior to the pandemic and, after further discussion, determined that virtual fitness might be less intimidating to patients than in-person wellness programming. During week 4 of the COVID-19 pandemic, staff implemented a wellness program that included 1-on-1 phone meetings with a personal trainer. Weekly fitness plans have successfully engaged patients in tracking their food intake, “cutting carbs” after lunchtime, and mixing up runs to include sprints and more mileage.

A month before the pandemic hit New Orleans, EPIC-NOLA began a consultation service for a new first-episode psychosis (FEP) clinic’s staff at 1 of the 10 local governing entities (LGEs) in Louisiana. EPIC-NOLA’s program manager has successfully maintained weekly consultation services via telehealth, including supervision and staffing. Monthly consultation on medication management also continued between EPIC-NOLA’s medical director and the LGE’s prescriber. Telehealth’s shared screen and “record” features have been useful for referencing training materials and sharing case presentations with trainees.

Before the pandemic, EPIC-NOLA served as a training ground for medical students from Tulane School of Medicine. Medical students have continued to work with staff on the clinic’s research and psychosis awareness campaign throughout the pandemic. The clinic’s first Master of Social Work intern, who began in January, also transitioned to the virtual clinic and is being trained and supervised via telehealth.

Despite the many successes achieved during the first wave of the COVID-19 pandemic, EPIC-NOLA has encountered several barriers in providing care. First, the initial transition to telehealth proved to be easier than the maintenance of an online clinic. Subsequently, EPIC-NOLA has created new policies and procedures for telehealth and virtual care.

In the initial transition to virtual care, staff acquired signed telehealth consent forms from all existing and new patients. The process of acquiring signed consents virtually was onerous and required multiple follow-ups by clinic staff. Barriers to obtaining signed consents included patients not having access to a printer and not being technologically familiar with, or cognitively well enough, to e-sign consent forms. Acquiring completed and signed paperwork for new patients proved to be equally difficult. Incomplete paperwork became clinically relevant as some patients were not completing release of information forms (ROIs), which prohibited clinicians and physicians from accessing complete medical records and fully understanding patient history upon intake. Acquiring signed ROIs and requesting medical records after patients have been discharged from the care of others have resulted in an endless game of chase. In response to these administrative challenges, and in preparation for the second wave of the pandemic, clinic staff may require all new patients to present to the clinic to sign paperwork in person, or staff will use a HIPAA-compliant application that eases completing and signing documents online.

EPIC-NOLA’s hospitalization rate remained steady with a non-significant increase from 11% to 13% (still well below the national average of 34% for first-episode programs) during the first wave of the COVID-19 pandemic. A total of 18 individuals were hospitalized, and 5 individuals had 2 or more hospitalizations. The relatively stable hospitalizations was not surprising given trends in decreased emergency room visits and non-COVID-19 hospitalization.^9^ However, the quick release of patients from hospitals and the subsequent number of re-hospitalizations were unexpected. This increased incidence proved challenging to manage clinically. Hospital staff were strained given COVID-19 protocols and were not as communicative with clinic staff as they previously had been. Additionally, discharge papers and ROIs proved difficult to manage when records were not sent in an efficient manner. In response to these challenges, EPIC-NOLA designated the clinic nurse as the contact person for all hospitalizations, the person responsible for documenting admissions and discharges in the electronic medical records, and the person reporting to the team at daily huddles. To improve engagement and reduce hospitalizations during a second wave of COVID-19, clinic staff will also encourage patients (especially new patients) to use the 24-hour on-call telephone for managing the intrusive symptoms of psychosis that can lead to hospitalization.

Finally, clinic systems disintegrated in the absence of having a physical front desk. In the virtual clinic setting, staff were responsible for checking patients in and out, the front desk had no contact with patients, and co-pays were forced to be collected at the end of the clinic day. The front desk especially had difficulty reaching patients and gathering co-pays. In the virtual setting, patients seeing the doctors for medication management were not walked to the front desk for follow-up appointments. Consequently, patient follow-up appointments were not scheduled, and no-shows were overlooked. Moving forward, co-pays will be collected prior to sessions, and doctors will schedule visits themselves to prevent loss of follow-ups.

## Conclusion

In the context of a global pandemic, the EPIC-NOLA made a rapid and successful transition to telemental health for a vulnerable population. The transition to and level of engagement in telepsychiatry and remote psychotherapy offer other FEP programs ideas about how to further integrate telemental health into CSC and engage patients during a public health crisis. Expanding telemental health programming and recalibrating administrative systems have the potential to improve clinical engagement, mitigate symptoms, and prevent repeated hospitalizations during another wave of the COVID-19 pandemic.
