# Psychosis, Telehealth, and COVID-19: Successes and Lessons Learned From the First Wave of the Pandemic – Erratum

**DOI:** 10.1017/dmp.2021.299

**Published:** 2022-10

**Authors:** Serena Chaudhry, Ashley Weiss, Grinasha Dillon, Ariana O’Shea, Tonya Cross Hansel

**Keywords:** community mental health, mental disorders, pandemic, erratum

Due to an oversight in the proof correction process, the hospitalization statistics in Table 2 of this article^1^ are incorrect and do not match the narrative. The correct hospitalization statistics are 11% and 13% respectively. The corrected table is presented below.

The publisher apologizes for the error.

**Table 2.** Overall clinical engagement with EPIC-NOLA (pre-COVID-19 vs COVID-19)

Variable	Pre-COVID-19 March 16 to May 15, 2019 (%)	COVID-19 March 16 to May 15, 2020 (%)
Total patients seen Show-rates	107	137
Total appointments	67.47	72.18
Physician (MD) show-rate	64.73	58.51
Therapist show-rate	68.67	83.84
Hospitalization	11.00	13.00
Race		
African American (83)	59.66	59.63
Caucasian (41)	29.64	33.76
Hispanic (10)	7.32	5.50
Vietnamese (2)	0.37	0.55
American Indian (1)	3.00	0.55
Gender		
Female	29.99	29.99
Male	70.07	70.07
Engagement		
Client’s engaged > 2 years	45.40	62.20
Client’s engaged < 2 years	54.60	37.80
